# The Literature of Dental Mechanism

**Published:** 1881-02

**Authors:** 


### ARTICLE II.

### *The Literature of Dental Mechanism.*

BY PROF. HODGKIN.

The volume before us * is the only American book on Dental Mechanism, or as Prof. Richardson prefers to term it, Mechanical Dentistry. It has stood for years as a text book in the dental schools, and, excepting the excellent division on this subject in "Harris Principles and Practice," by the late Prof. P. H Austen, has no competition. The publishers have lately brought out a new edition of this work, containing many new things, some perhaps more properly belonging to Dental Surgery than to Mechanism, as these subjects are commonly classed ; and the volume is thus increased in size considerably.

The revision of an old book would seem a more difficult task than its original composition. At least so we take it in looking at the work of this sort which is done from time to time. Whether a volume after being accepted as a text

*"A practical treatise on Mechanical Dentistry, by Joseph Richardson, D. D. S., M. D., Prof. in the Indiana Dental College, etc. Third edition, revised and enlarged. Philadelphia, Lindsay & Blakiston 1880."

book acquires a sort of sanctity which is in itself inhibitory, or whether it is that the writer feels loth to change what is once written; or, whether authors, like other people, hesitate to change, lest they should be suspected of weakness, it is difficult to say. But it does seem as though that plasticity of opinion which in the rapid advance of dental and other sciences leads to new views and modification of old views, is not a characteristic of revisers. We are compelled usually to go to the new *books*, not to the new editions of the old books, for the latest light. True it is that text books such as this are of necessity behind the times, for even the most rapid compilations and recent condensations from journalistic literature grow musty and are left behind, whilst the volume is yet in the hands of the printer and binder; and the works become, even in the present, a thing of the past.

Yet this should not excuse a book-maker from the most rigid scrutiny of what is already written and which has stood before the public as an index of truth, to guide seekers after knowledge; and when a new edition is in progress, it is not enough simply to *add to* what is already printed. Expurgation and emendation is no less the duty of the editor—often the most important duty—than addition; and in all fairness to Prof. Richardson, this we claim, and hope to show, he has not done. Of course it is easy to criticise and find fault. We only hope to show that errors such as are made, are grave defects in a text-book, in which young men are searching for the best and most carefully thought out precepts and doctrines, and in which no information easily accessible should be incomplete or incorrect.

Somehow we cannot help feeling that Prof. Richardson has hardly made practical use of all which is put down in this book as useful. The description of the apparatus of Mr. Fletcher, of England, is evidently copied from the vauntings of that highly egotistical gentleman, who turns out from time to time things newer than his newest, only to find that his *last* is just the thing. Long ago, in a book most tersely written, and *par excellence*, full of original thought and original research—we may almost say the only original writer on these subjects—Prof. Austen suggested the addition of a flexible tube to the mouth blow pipe, and of a cigar-holder as a mouth piece. And he seems to have been the only man to give a philosophical description of the proper terminal point or tip of this instrument,—that it should terminate in a perfectly parallel-sided tube or cylinder, smooth and polished within; and he further suggests the straightening of the curved end, which Prof. Richardson claims, or allows Fletcher to claim, as original. It is not probable that an original investigator would fall into such apparently small errors as these; nor would one who examined and studied all things with the mathematical criticism of Austen have said, "it is *claimed* that with the use of this instrument," etc. We must say, even at the risk of being considered harsh, that in such matters only the personal investigations of the writer carry with them conviction—the "claims" of the inventor should be set aside, and only personal knowledge recorded; and this for obvious reasons.

For the benefit of young men, and the more especially in this day of "plastics," when a vulcanizer for artificial dentures, and a spatula for filling, constitutes an outfit, mechanical and operative, and when the blow-pipe is a purely theoretical instrument, it is worth while to say that the immense and urgent blast and heat of the new forms of apparatus are really not essential in soldering. No more beautiful soldering can ever be done in the future than has been done in the far past, with a simple mouth blow-pipe and an old fashioned oil or alcohol lamp. The most ingenious device will only supplement—not take the place of skill; and if this is acquired, even the indifferent and commonplace tools are given dignity and excellence; are capable of producing results little short of marvellous. When once the "how to do it" is acquired, simple means will ordinarily suffice.

It is unfortunate to refer as reference, to a book now out of print, as is done on page 40. Piggott's Dental Chemistry is unfortunately no longer purchasable; it is a book full of reliable information on such subjects as are referred to by Prof. Richardson; and no more valuable contribution to scientific study in dentistry could be made, would some one qualified re-write it up to the latest state or phase of chemistry.

It seems a singular omission that in the treatment of the subject of gold, its most commonly associated metal, silver, should be omitted. It is rarely absent in the gold mined from our chief sources, California and Australia; and the facts that this metal is very malleable, ductile, unoxydizable by heat, etc. are not those which "make it peculiarly fit for the purposes to which it is applied in dental prosthesis." It is the tested behavior of a substance *in the presence of the oral secretions*, which determines its fitness, or the reverse; and this, whether it be a filling or a plate. And with regard to the question of its malleability (p. 44) Makins is authority for saying that gold alloyed with copper or with silver (but not with both) is more readily beaten than the pure metal.

The chapter on refining gold is hardly sufficient by explicit, and a simple though important and effective method of separating the mechanically mingled impurities, and base metals, as well as silver scrap, is not noticed. Nitric acid will, in addition to dissolving out the iron sought to be rid of by the use of the magnet, take out all other mechanically mingled metals capable of solution in it, and by washing out the soluble nitrates, a simple means of preliminary purification is had. A magnet is apt to entangle filings of gold along with the iron it may attract. Mercuric chloride is, according to the best authorities, a wasteful salt for purification, as, along with the volatilized mercury is carried off an appreciable quantity of gold. It is never used by specialists; but chlorine gas, which Prof. Richardson does not mention, is used with great advantage to toughen gold.

Now that graphite crucibles are so largely used, it is proper to say that their use is not practicable in dry refining, this material hindering oxydation. The "wet process" for refining gold is fairly stated, but much more minute instructions would be necessary to enable a novice to perform this delicate and somewhat complex chemical process. In fact we have seen but one work by the aid of which a student could conduct these manipulations intelligently, and this is Makins' Metallurgy, an English work. No one who wishes to study metallurgy can dispense with this book. It is well to know, what is not mentioned in the book under review, that the troublesome accumulation of fluxes, glass of borax, etc. on the top of the molten metal in the crucible may be readily removed by the use of bone ash, which acts here as a sponge.

On the question of alloying gold plate, the student will be likely to draw the inference that copper is injurious, whereas the fact is it takes less copper to produce the requisite degree of hardness than of silver. Twenty carat gold plate in which the alloy is copper, will not tarnish in most mouths. But in many cases—more than may be supposed—gold coin of the U. S. may be used with advantage. But of the large array of disorders cited by Prof. Richardson as the sequence of wearing a plate of low carat value, it is sufficient to say that where "indigestion, with acid eructations; gastro-enteritis, ague, inflammatory and typhoid fevers; brain affections, eruptive diseases; rheumatism, gout, etc." exist, that the "natural sequence" of one or all of those affections would be to produce the troubles he charges to the corroded plate, viz: "inflammation of the mucous membrane and gums, with chronic periodontitis and loosening of the teeth; apthous ulcers; gastric irritations, general nervous disorders; decay of the teeth, fœtid breath; disagreeable metallic taste in the mouth, etc." It would be almost as fair to charge the plate with the gastro-enteritis, ague, gout, etc., as with the local diseases which are the common sequelæ of such grave diseases, and which are thus given such obviously wrong etiology. It is such blunders as these that have brought upon dentistry the stigma which the medical profession attach to us, of would-be scientists, and if this is taken as a sample, justly.

On the subject of clasps, no mention is made of the alloy of platinum and iridium, which is far superior to the alloy of gold and platinum for this purpose. And indeed this alloy, so useful for various operations in dentistry, in the shape of wire for pivoting, etc., as to be well nigh indispensable to the writer, is not mentioned in the chapter on alloys as at all useful. Moreover, Prof. Richardson seems to be oblivious of the fact, as he does not mention it, that the alloy of gold and platinum is so readily tarnished as to be quite objectionable on that account; much more readily than the alloy of gold and copper.

It is well to mention, what is omitted in this work, that there are two guage plates of different size, in common use—the American, and Stubb's. A lack of this knowledge may lead to confusion; and also that small quantities of solder may be quickly and readily made on a piece of charcoal, this latter substance greatly preventing the oxydation of any oxydizable metals present. A simple method of obtaining pure copper for alloying purposes would be valuable also, in this place.

On the subject of the removal of roots of teeth, as preparatory to the insertion of an artificial denture, it is said by Prof. Richardson, that the roots of teeth, if allowed to remain, offer "so many fixed points of resistance," and the mucous membrane yielding, allows the piece to rest or press unduly on the former. But, unless there be only a few roots, these, if healthy, should bear the pressure of the plate as well as the teeth formerly supported by them, and of which they were parts, sustained the pressure of mastication. The truth being that, on account of the inevitable change in the upper lip and its muscles, due to absorption of the alveolus, *and which no artificial appliance can fully*

*restore*, it is highly important to leave, whenever it can be done, the roots of the six anterior superior teeth in position, provided these can be restored to health, if diseased. Of course in all cases they should be so filled as to prevent the recurrence of decay. An artificial appliance can be made of perfectly satisfactory character for the mouth of any such patient; and it is the duty of the dentist to know how to do this, and not extract these roots, to simplify his work.

It is an unphilosophic and incomplete book which passes over the subject of the changes which occur in the jaws after removal of the teeth, in the manner of the one under review. No attempt is made to explain the rationale of the process of absortion; no mention made of the changes in facial conformation, displacement of muscles, etc; and nothing is said of how important a factor pressure is in hastening, modifying, and, possibly retarding absorbtion. It seems singular that so obviously an important branch of this subject should have escaped the attention of the author.

The chapter on wax impressions is faulty in its lack of detail, and especially in that it should have given importance to thoroughly cooling the wax before attempted removal from contact with the ridge; and no teaching on this subject is complete which neglects the cardinal principle that *a thin layer of impression material* is essential to correctness in copying. No mention is made of the chemical constitution of plaster, or of the fact that it is necessary to calcine it before using it, and neglect is made to state that where the setting of plaster is hastened by the use of salt, that such a cast is worthless except for almost immediate use. The use of potassic sulphate, so superior to sodium chloride for hastening the setting, in that it does not injure the plaster, is not mentioned. One would judge from the remarks of the writer, that he was unacquainted with the value of plaster as an impression material in partial cases, but this part of the subject is mixed up so with various methods quoted that it is difficult to judge how much weight Prof. Richardson attaches to his own views. No mention is made, or description given of Bean's admirable method of swaging a cup and coating with shellac and cotton, for plaster impressions. In difficult cases it is by far the most accurate method known to the writer of attaining a perfect impression. No one who has once used a small curved spatula for dilating the mouth, while introducing the cup, will ever dispense with this useful appliance. This Prof. Richardson does not seem to know of, or does not mention its use. Prof. Austen's admirable invention of an improvised gutta percha cup, is not mentioned at all. In reference to gutta percha, its property of contracting is referred to as rendering it necessary to fill up the impression immediately with the plaster cast. But as most, if not all of this cooling and contraction would occur before the cast solidified, it would seem that this process was unphilosophic. Prof. Richardson expresses the opinion in his book that it is impossible to get a correct impression in wax for a full denture, and we gather from the tone of the book that he likewise deems it difficult, if not impossible, to take partial impressions with plaster. Most dentists will be apt to reverse these opinions. No recognition is given of the importance of filling the impression, especially if it be of a partial case, with water to facilitate getting the plaster into the cavities in the same; or of tapping the cup to settle the soft plaster.

On the subject of pivoting teeth Prof. Richardson remarks, in speaking of cases where constitutional tendencies to inflammation exist, that "the operation, if done at all, should be done in the most careful manner." We respectfully suggest that any operation, under any circumstances, should be performed in the most careful manner, and he who in any case neglects this, is gravely at fault.

In preparing the root, the use of drills is accompanied with the absolute injunction that "they should be kept wet," while the almost universal practice at present is to use drills and burs with the cavity dry, and the rubber dam adjusted. No mention is made of Gates' drill, so invaluable in reaming out the canal. The chapter on pivoting is quite long and compiled largely from recent sources of information, though much of it is dental surgery proper, as much so as filling teeth. Bonwill's latest methods for pivoting are given in detail, methods not really so good as his earlier devised platinum pin and nut.

In regard to those front root amalgam fillings, we have found that in all cases, noticed so far, a disagreeable blue hue is given to the gum by the discoloration of the root by the amalgam.

The chapter on swaging is faulty, in not being sufficiently explicit and full. Thus the use of a jeweller's saw for cutting the plate to shape is not mentioned, and there seems to be no knowledge of the fact that plates, either partial or full, should be stamped nearly to shape before trimming ; and what is even worse, no mention is made of the dangers that attend annealing a plate which has been swaged between lead and zinc, without preliminary careful cleaning in acid to rid the gold of any possible contamination. No means seems be to known to Prof. Richardson whereby pleating or folding of a plate may be prevented : and, helpless in the presence of this easily prevented accident, advice is given to cut out the fold and solder the joint. No mechanician who took a pride in his work, and who thoroughly understood swaging would tolerate such a procedure. Doubling the plate is mentioned, but the method not described. The obsolete method of cylinders with wood contained, to press against teeth, for retaining partial pieces; as well as the pivoting of a plate with several teeth to roots, are both retained, as though still in use. Neither the philosophical principles underlying the use, or the common abuse of vacuum cavities are mentioned at all, nor the injurious effects of the deep cavity alluded to ; while the important subject of securing a correct articulation in partial cases, is dismissed with the observation that the plate, with a rim of soft wax on it, is to be placed in the mouth, and the patient instructed to close the jaws naturally. No mention is made of the desirableness, in cases where there are three or more points of articulation, of taking an impression of the lower teeth, procuring a cast, and using the upper and lower casts thus obtained to determine the bite ; a much more exact, convenient and elegant method than any other. In taking bites in full cases, Prof. Richardson gives only the impracticable method, by which no one ever controlled the erratic movements of the lower jaw, of pressing it backward with the hand. True, some pages further on, the remark of Dr. Bonwill is quoted that the act of swallowing will prevent protrusion of the jaw, but this is not grouped with the directions in proper place. It may be said that these are captious objections, and that it is a small matter. The whole of dentistry is made up of small matters. Over five pages is given to the description of the "anatomical articulator" of Dr. Bonwill, of which we have only to say that as Dr. Bonwill states, and truly, that "if the bite in wax is not correct, *no articulator can make it so;* you must go back to the mouth and retake it,"—the common backward extension of the model in plaster will serve the purposes of articulating as perfectly and more conveniently than anything else. In grinding porcelain teeth, no instructions are given to trim the plaster cast when plain teeth rest directly on the natural gums, (in metal plate work) to secure neatness of adaptation to the gum; and nothing is said of corundum wheels, as to the care that should be taken of them, methods of dissolving of the gum upon the surface where the wheel may cut badly from heating; and no instructions as to how to mount wheels. In fact *only three lines* is given to this subject. The subject of "carved block teeth" occupies nineteen pages. It may be well to keep this in a "book up with the times," but it is hardly probable that many will study this almost lost art, or art superseded by special manufacturers. We suggest that a carefully prepared compendious description of the continuous gum process would be better than the rather diffuse statements of five several writers. On the subject of vulcanite, Prof. Richardson risks the perdiction that the abandonment of this substance "as a base for artificial dentures by intelligent and conscientious practitioners is an event of the near future," and he thinks this "would be in the interests not only of the profession, but of all concerned." Many imperfections and omissions might be noticed in the chapter on vulcanite, but we have no time to notice these, and this chapter is one of the best in the book. We remark as noticeable the absence of any method of repairing vulcanite plates, of the use of a solution of vulcanizable gum in benzine or other solvent as a cement between the new and old portions of the plate. He will find few, however, to agree with him in his statement that the "chief objection to celluloid is its low power of transmitting caloric." A full chapter is given to Dr. Reese's gold alloy cast late, while no mention is made of the old and well received stanno-plastic methods of Wood and Weston. That these are useful is beyond question, and time has tested them, which cannot be said of the method of Reese. This latter, however, would answer well for the alloys above mentioned.

Dr. Kingsley has a chapter on the treatment of defects of the palate, in which the soft rubber velum is insisted upon. But if we remember rightly, Dr. Kingsley has lately stated that a hard rubber velum is as efficient; while Dr. T. B. Gunning, no mean authority, states that the hard rubber is the *only* efficient velum, fulfilling all the requirements of any case.

We have no desire to make a captious or fault-finding criticism of this or any book. It has many excellencies; but its defects are too obvious to escape the eyes of the teacher or trained reader. We have hinted at the cause of some of these defects, and feel them to be due to a lack of practical knowledge on the part of the compiler. We use this word compiler designedly, not knowing Prof. Richardson. It is apparent that the book is mainly a gathering together from the literature of the past years of dentistry, such material as seemed popular, without thorough individual testing of any of it, as to merit or usefulness. We feel sure that had the writer set himself as earnestly to work as did Prof. Austin to sift for himself every statement, analyze each alleged fact, and bring to the test of practical experience every step in his work, the book before us would have been free from many of the flaws we have pointed out. It is a striking commentary on the paucity and meagerness of dental literature that the one book written solely on Dental Mechanism, (the little publication of Mr. Coales, of England, does not deserve the name of a book,) should be so full of error and omission. It is to be hoped that this department of our profession will be written up. It deserves it.

